# Relative Age Effect in Canadian Hockey: Prevalence, Perceived Competence and Performance

**DOI:** 10.3389/fspor.2021.622590

**Published:** 2021-03-04

**Authors:** Jean Lemoyne, Vincent Huard Pelletier, François Trudeau, Simon Grondin

**Affiliations:** ^1^Département des Sciences de l'activité Physique, Université du Québec à Trois-Rivières, Trois-Rivières, QC, Canada; ^2^Laboratoire de Recherche sur le Hockey de l'UQTR, Université du Québec à Trois-Rivières, Trois-Rivières, QC, Canada; ^3^École de Psychologie, Université Laval, Québec, QC, Canada

**Keywords:** relative age effect, ice hockey, perceived competence, minor hockey, junior hockey, attitudes, performance

## Abstract

The term “relative age effect” (RAE) is used to describe a bias in which participation in sports (and other fields) is higher among people who were born at the beginning of the relevant selection period than would be expected from the distribution of births. In sports, RAEs may affect the psychological experience of players as well as their performance. This article presents 2 studies. Study 1 aims to verify the prevalence of RAEs in minor hockey and test its associations with players' physical self-concept and attitudes toward physical activities in general. Study 2 verifies the prevalence of the RAE and analyzes the performance of Canadian junior elite players as a function of their birth quartile. In study 1, the sample is drawn from 404 minor hockey players who have evolved from a recreational to an elite level. Physical self-concept and attitudes toward different kinds of physical activities were assessed via questionnaires. Results showed that the RAE is prevalent in minor hockey at all competition levels. Minor differences in favor of Q1-born players were observed regarding physical self-concept, but not attitudes. In study 2, data analyses were conducted from the 2018–2019 Canadian Hockey League database. Birth quartiles were compared on different components of performance by using quantile regression on each variable. Results revealed that RAEs are prevalent in the CHL, with Q1 players tending to outperform Q4 players in games played and power-play points. No other significant differences were observed regarding anthropometric measures and other performance outcomes. RAEs are still prevalent in Canadian hockey. Building up perceived competence and providing game-time exposure are examples of aspects that need to be addressed when trying to minimize RAEs in ice hockey.

## Introduction

The term “relative age effect” (RAE) is used to describe a bias, evident in the upper echelons of youth sports, where participation is higher among those born at the beginning of the relevant selection period—and, therefore, lower among those born at the end of the selection period—than would be expected from the standard distribution of live births (Larouche et al., 2010). In general, in the literature on the RAE, live birth distributions are analyzed in the form of quartiles (Q) in which birth months are grouped in four 3-month categories (Q1: January 1st–March 31st; Q2: April 1st–June 30th; Q3: July 1st–September 30th; Q4: October 1st–December 31st). Past research has shown that the RAE is predominantly found in many sports (Grondin et al., 1984; Turnnidge et al., 2014). This effect is mainly found in sports in which physicality prevails—such as soccer, rugby, and ice hockey (Lames et al., 2008; Till et al., 2010; Helsen et al., 2012)—and among male athletes (Romann et al., 2018). Ice hockey is a typical example where the RAE seems to be highly prevalent among male players (Barnsley et al., 1985; Wattie et al., 2007; Nolan and Howell, 2010) and female Canadian (Weir et al., 2010; Hancock, 2017), Czech (Bozděch and Zháněl, 2020), and Swedish players (Stenling and Holmström, 2014). Recently, an analysis of the 2017 International Ice Hockey Federation (IIHF) Championships showed that the RAE was prevalent mostly among goaltenders and forwards (Bozděch and Zháněl, 2019). RAEs were also found among Russian hockey players, who evolve in hockey academies and the *Kontinental Hockey League* (Bezuglov et al., 2020). In the province of Quebec (Canada), RAEs were also found at the minor hockey level, where the effects remained even after changes in cutoff dates for each age-group category were implemented (Lavoie et al., 2015).

Theoretically, RAEs are the result of multiple interactions involving individual, task-related, and environmental factors (Wattie et al., 2015). More specifically, this means that the development of RAEs will depend on variables such as participants' age (individual), the popularity of the sport (environmental), and multiple facets related to the sport itself (skills, positions). In Canada, ice hockey is one of the most popular sports, if not the most popular. In that sense, RAEs seem likely to occur. According to Hancock et al. (2013b), RAEs are developed through social interactions that involve 3 types of social agents: the players themselves (children and adolescents), coaches, and parents (in the case of minor hockey). Such interactions have the potential to influence social agents' perceptions about the players (and the perceptions of the players themselves), which could affect (either positively or negatively) the participation of children and adolescents in organized sports. From this perspective, if the RAE can considerably affect players in team selection at different levels, it might also be associated with psychosocial outcomes that would tend to favor early-born players (Q1, Q2). For example, the team selection process closely contributes to these RAEs. Early-born players are frequently perceived as more talented by coaches and are prioritized at the moment of player selection. Scouts, coaches, and parents are somewhat more impressed by these “more dominant” players, which leads to favorable outcomes for these players. Such attention toward their performances may reinforce early-born players' sense of accomplishment, which consequently can enhance their level of confidence in sports. These athletes will feel confident about their hockey abilities and competencies. In parallel, interactions involving players with coaches and parents have the potential to make the experience of a hockey player's progression a more enjoyable experience (e.g., the hockey experience is more fun). Inversely, late-born (e.g., Q3 and Q4) players, who are more likely to be rejected from teams early in their “hockey career,” live the opposite scenario: coaches will perceive them as not strong enough, or “not ready for the next level.” Indeed, late-born players may feel that they are losing the opportunity to play at a higher competition level, which can potentially hinder their sense of accomplishment and make their hockey experience less enjoyable. Consequently, in front of what could appear as a “wasted” season, late-born players may decide to stop practicing their sport earlier in the process. RAEs were also demonstrated in education, where early-born primary school girls (Sprietsma, 2010) and adolescents (Cobley et al., 2009) tended to get enrolled in higher-performing academic programs and to obtain higher scores in different subject areas. RAEs were also reported in physical education classes, with higher motor skills among early-born pupils (Gadžić et al., 2017). Fenzel (1992) also showed that early-born students had higher self-esteem and tended to perform better in school. In sports, RAE research showed that early-born players get more exposure and are frequently perceived as more talented or physically “gifted” than their younger counterparts (e.g., late-born players; Hancock et al., 2013b). From this perspective, such exposure, and the interactions that result from this, should affect on psychological outcomes such as self-esteem (Fenzel, 1992) and perceived sport competence (Guillet et al., 2002). As shown by Kawata et al. (2017), early-born players (e.g., Q1) might gain psychosocial benefits such as increased enjoyment and more favorable attitudes toward physical activity. On the other hand, such an uneven way of selecting young athletes may have negative consequences on their desire to pursue their athletic careers (Musch and Grondin, 2001). By reducing their chances to access elite status, young athletes may become discouraged, eventually leading to sports attrition (Musch and Hay, 1999; Vaeyens et al., 2005). It is reasonable to posit that late-born players must have a less enjoyable hockey experience. Despite the contribution of past research to understanding the mechanisms underlying RAEs in sports (Baker and Logan, 2007; Baker et al., 2010), no recent data are available regarding RAEs among minor hockey players in Canada, a country counting more than 600,000 registered players in organized hockey (Hockey Canada, 2019).

The RAE may also exert its influence on different aspects of performance. Despite the early advantages that RAE affords players in multiple facets, some authors suggest the possibility of a reversal of RAEs on a longer-term basis (Gibbs et al., 2012). Reversal of RAEs is defined as a period in which late-born players (Q4) tend to catch up with, or even outperform, early-born players at higher levels of competition. Two hypotheses could explain the reversal of the RAE in sports. The first hypothesis is psychological, which stipulates that younger players tend to develop a better capacity to adapt by getting the opportunity to develop psychological attributes such as increased motivation, resilience, and capacity to overcome challenges. This psychologically related hypothesis was tested in elite soccer (Ashworth and Heyndels, 2007) and should also apply in the context of ice hockey. Like professional soccer, the pathways toward elite or professional hockey are paved with many obstacles that can be overcome with a high degree of resilience, motivation, and determination. A good example of the importance of such factors comes from Herbison et al. (2019), who underlined the importance of considering competitiveness, passion, and confidence as factors associated with the capacity to overcome obstacles among undrafted NHL players. Under such circumstances, late-born players who evolved in positive and favorable environments are potentially less affected by RAEs, which means that their social environments (coaches, parents, and teammates) may interact favorably toward the development of these psychological assets. In complementarity to this first hypothesis, the biological–athletic reversal effect occurs among those who have the genetic and athletic background that predisposes young athletes for excellence (Ashworth and Heyndels, 2007; McCarthy et al., 2016). Support for the biological–athletic hypothesis was obtained at the National Hockey League (NHL) level. A study published by Fumarco et al. (2017) showed that despite being drafted later, late-born players (Q4) tend to be more productive and durable than players who were preferred in the past due to their birth date (Fumarco et al., 2017). However, no study has yet to allocate specific attention to the moment of occurrence of this pattern. In this regard, it is relevant to try to shed light on a possible fading of the RAEs by verifying if it occurs at a younger level such as the Canadian elite junior level.

### Objectives of the Study

This article includes two studies related to the RAE in Canadian hockey. In study 1, the focus is on a sample of young (12–17 years old) minor hockey players, and the aim is to verify a RAE on outcome variables such as attitudes toward sport and perceived competence in hockey. The study also verifies whether the RAE differs according to the playing levels of young players. For study 1, our hypotheses are based on previous research and on the potential impacts of RAEs, suggesting that early-born players (Q1–Q2) should display more favorable-positive attitudes toward sport and should show a higher level of perceived competence in the physical self, compared to late-born players. Taking into account various other factors such as age groups and competition level (on the distribution of births and psychosocial outcomes) should make it possible to further analyze how RAEs influence players throughout their hockey progression as a player.

Study 2 aims to verify the prevalence of the RAE and its relationships with performance indicators in the Canadian Hockey League (CHL), which regroups the best junior players in the country. Our first hypothesis for study 2 is based on recent ice hockey research (Nolan and Howell, 2010; Bezuglov et al., 2020) and posits that even 35 years after the results from Barnsley et al. (1985) and Grondin et al. (1984) with players from the CHL, RAEs persist. A second objective of study 2 is to determine whether RAEs translate into different levels of performance in Canada's top junior league. A lack of significant differences (in performance outcomes) between players born in the different quartiles would suggest a possible reversal (or fading) of RAEs at the CHL level, which would be in line with the results of Fumarco et al. (2017).

## Study 1: Prevalence and Psychosocial Outcomes in Minor Hockey

### Sample

The sample in study 1 consists of 404 young hockey players aged between 12 and 17 (15.4 ± 1.9 years). All players were registered with Quebec's provincial federation and came from 3 different age groups based on the provincial federation's categorization guidelines: U13-Peewee (11–12 years old: *n* = 95), U15-Bantam (13–14 years old: *n* = 63), and U17-Midget (15–17 years old: *n* = 246). Players also came from two competition levels [competitive/elite (*n* = 202; 50%) and recreational (*n* = 202; 50%)]. Competitive level players are registered in the *Ligue de Hockey d'Excellence du Québec* (LHEQ) and play the AA and BB levels. These players represent their region and generally have a higher training volume (2–3 training sessions per week) and more demanding schedules (35–50 games per year). Recreational level players are those who play at the community level (house league); they train less often (e.g., once a week) and play fewer games (25–40 a year). We used a convenience sample and tried to replicate the proportions of participants at each level of competition in Quebec. The project was approved beforehand by the ethics committee of the lead researchers' academic institution (CER-17-240-08-01.10) and by the provincial hockey federation (Hockey Québec). We implemented 2 data collection protocols. First, we conducted an initial data collection during the teams' meetings following one of their training sessions. Fifteen teams at the competitive/elite level, of which nine agreed to take part in the study, were randomly selected on the Hockey Québec website. We then contacted coaches by phone to obtain their permission to meet and inform their players about the research. For participants under 14 years old, a parental written consent was required. Once the coaches agreed to participate, the research team handed out questionnaires to the participant, who was previously placed in a quiet room to ensure their concentration. For the second protocol, we collected data directly during hockey tournaments. To this end, we obtained tournament directors' endorsement a few weeks beforehand. The research team approached 5 tournament directors and three accepted to take part in the study. Subsequently, we asked the randomly selected teams' coaches for permission to meet and inform their players about the project and to distribute the questionnaires. To avoid interfering with the tournaments and to ensure the players' full concentration, they had permission to take the weekend to fill out the questionnaires.

### Measures

Data came from a questionnaire used in a study designed to investigate the environment of adolescent hockey players, which included environmental and psychosocial variables [for a detailed description of the questionnaire, see Huard Pelletier et al. (2020)]. This questionnaire includes environmental factors such as accessibility and opportunities for sport, social factors related with coaches and parents, and two psychosocial constructs: attitudes and physical self-concept. In the present study, the first part of the questionnaire assessed some sociodemographic variables and birth date. Each birth date was coded into birth quartiles (Q1 to Q4) by referring to the usual categorization that is used in each league: (1) Q1: January–February–March, (2) Q2: April–May–June, (3) Q3: July–August–September, and (4) Q4: October–November–December. For the second part of the questionnaire, we used two specific psychosocial outcomes, namely, attitudes toward sport and exercise (hockey, sport, weight training, and aerobic exercises) and physical self-concept measures (physical self-concept, perceived sport competence, perceived endurance, and perceived strength).

***Attitudes*** toward sport and other exercise behaviors were measured using a 12-item semantic scale. We measured attitudes toward two categories of behaviors: (1) hockey-sport (ice hockey: 3 items; leisure-time sports: 3 items) and (2) exercise behaviors (strength training: 3 items; cardiovascular exercises: 3 items). We asked participants to rate their level of agreement on a six-point scale (low score = negative attitude, high score = positive attitude) by completing the following sentence: “For me, [playing ice hockey] is [useless-useful, unpleasant-pleasant, demotivating-motivating].” Preliminary analyses showed satisfactory reliability for each subscale, with excellent McDonald's omega coefficients (ω_Hockey_ = 0.89, ω_sports_ = 0.88, ω_strength_ = 0.94, ω_cardio_ = 0.89). From each subscale, we calculated two composite scores for attitudes.

***Physical self-concept*** was measured with the Physical Self-Description Questionnaire (PSDQ: Ninot et al., 2000). Thus, 3 dimensions (subscales) of physical self-perception were measured, using 12 items: perceived sport competence (4 items), perceived cardiovascular endurance (4 items), and perceived physical strength (4 items); each subscale was conceived as a six-point Likert scale (from 1 = unfavorable perceived competence to 6 = high perceived competence). We also calculated a global self-concept score by using all 12 items. Preliminary analyses show a very good reliability for each subscale (ω_Sport_ = 0.87, ω_Strength_ = 0.85, ω_Endurance_ = 0.83, ω_global_ = 0.79). Then, we used the 3 subscales, plus a composite score (mean score calculated from the 12 items), for a global measure of physical self-concept.

### Statistical Analyses

We verified for the prevalence of RAE by conducting cross-tabulations (chi-square statistic) on players' birth quartile distribution. To prevent bias in the distribution of birth quartiles, we followed the recommendations of Delorme and Champely (2015) and compared the birth rate of Québec in 2004 (the vast majority of players of the sample were born in Quebec), which corresponds to the median age of our sample (provincial birth rates: Statistique Canada, 2018). In line with Delorme and Champely (2015), we also tested each age category with its previous group, to confirm if birth distributions differ from one category to another. This resulted in 3 group comparisons: U13 vs. U15, U15 vs. U18, and U13 vs. U18. Significant differences at this level would mean that RAEs would affect sport participation regarding age groups. For the second part of our analyses (psychosocial outcomes), we conducted nonparametric group comparisons to compare birth quartiles regarding attitudes and physical self-concept scores and to evaluate if early-born players (Q1 and Q2) displayed more favorable attitudes toward physical activities and a stronger physical self-concept. We used the Kruskal–Wallis *H* and the test on median scores to test for significant group differences.

### Results

#### RAE Among Quebec's Minor Hockey

Table 1 shows that birth distribution differs regarding birth quartiles and is significantly different from the numbers obtained from the national survey (Statistique Canada, 2018), with players from Q3 and Q4 representing only 41% of the total sample (χ^2^ = 12;593, *p* < 0.01). Therefore, our data showed that RAE is prevalent in minor hockey in Quebec.

**Table 1:** Birth quartiles in minor hockey compared with 2004 live births (Statistics Canada).

**Birth quartile**	***N* [Standardized residuals]**	**%**	**Birth quartiles—Quebec 2004 [standardized residuals]**	**χ^2^**
Q1	123 [23.5]	30.4	24%	
Q2	110 [10.5]	27.2	25%	12.593^*^
Q3	85 [−14.5]	21.0	26%	
Q4	80 [−19.5]	19.8	24%	*W* = 0.178
Missing	6	1.5	N/A	
Total	404	100%	N/A	

* *p < 0.01*.

#### Age Group, Playing Level, and Category

Results showed that, for age groups (Table 2), birth quartile repartition is similar across age groups (χ^2^ = 4.42, *p* = 0.62; Cramer's *V* = 0.08, *p* = 0.62). When we compared each age category with the previous one, these differences remained non-significant (all *p* > 0.25). Similarly, no RAEs related with playing level were observed (χ^2^ = 1.82, *p* = 0.61; Cramer's *V* = 0.07, *p* = 0.69), suggesting that despite the observed disproportion of births in quartiles, the magnitude of the RAEs in the different playing levels remains essentially the same.

**Table 2:** RAE prevalence across age groups and playing levels.

**Birth quartile**	**Age groups** ***N*** **(%) [Standardized residuals]**			**χ^2^**	**Playing level** ***N*** **(%) [Standardized residuals]**		**χ^2^**
	**U13**	**U15**	**U18**		**Recreational**	**Competitive**	
Q1	39 (31%) [7.5]	39 (31%) [7.3]	44 (31%) [8.3]	4.42^ns^	64 (33%) [15.8]	58 (29%) [7.8]	1.82^ns^
Q2	28 (22%) [−3.5]	40 (32%) [8.3]	41 (28%) [5.3]	*W* = 0.11	49 (25%) [−0.2]	61 (30%) [10.8]	*W* = 0.07
Q3	29 (23%) [−2.5]	23 (18%) [−8.7]	33 (21%) [−2.7]		44 (22%) [−5.2]	41 (20%) [−9.2]	
Q4	30 (24%) [−1.5]	25 (19%) [−6.7]	25 (20%) [−10.7]		39 (20%) [−10.2]	41 (20%) [−9.2]	

*ns, non significant*.

#### RAE on Psychosocial Outcomes Among Minor Hockey Players

Table 3 shows descriptive statistics on attitude scores and measures of self-concept for each birth quartile. Results from Table 3 indicate that there are no significant differences between quartiles due to a RAE for attitudes toward sports and hockey (*H* = 4.51, *p* = 0.21) and attitudes toward exercise behaviors (*H* = 0.80, *p* = 0.85). Most participants showed strong and favorable attitudes toward physical activities, and these attitudes are similar in the different quartiles. No significant RAEs were observed on attitudes regarding age groups and playing level (all *p* > 0.30).

**Table 3:** Comparisons of birth quartiles regarding physical self-concept and attitudes toward physical activities.

	**Median (Md)**	**Q1**	**Q2**	**Q3**	**Q4**
Attitudes toward hockey and sports	5.75	5.47 ± 0.72	5.61 ± 0.43	5.48 ± 0.65	5.58 ± 0.59
*N* > Md (%)	N/A	49 (41%)	50 (45%)	34 (40%)	30 (38%)
Attitudes toward exercise	4.75	4.67 ± 1.00	4.60 ± 0.98	4.51 ± 1.18	5.53 ± 1.04
*N* > Md (%)	N/A	60 (50%)	53 (48%)	43 (50%)	39 (51%)
Physical self-concept	4.42	4.59 ± 1.14	4.38 ± 0.62	4.28 ± 1.04^*^	4.58 ± 1.47
*N* > Md (%)	N/A	66 (67%)	60 (55%)	29 (35%)	37 (47%)
Perceived sport competence	5.00	4.94 ± 0.95	5.01 ± 0.78	4.67 ± 0.92^**^	4.81 ± 0.95
*N* > Md (%)	N/A	53 (48%)	54 (49%)	28 (33%)	34 (45%)
Perceived strength	4.25	4.38 ± 1.42	4.13 ± 1.09	3.92 ± 1.04^***^	4.05 ± 1.09
*N* > Md (%)	N/A	58 (49%)	53 (48%)	27 (33%)	30 (39%)
Perceived endurance	4.33	4.27 ± 0.95	4.27 ± 0.75	4.06 ± 0.99	4.20 ± 1.17
*N* > Md (%)	N/A	47 (39%)	33 (30%)	22 (27%)	28 (37%)

* *Q3 < Q1: H = 9.085, p = 0.028*.

** *Q3 < Q1: H = 7.196, p = 0.066*.

*** *Q3, Q4 < Q1: H = 7.117, p = 0.068*.

*N > Md: proportion of participants (%) who reported a score higher than the median score*.

For the physical self-concept measures, RAEs were observed in general self-concept, with Q3-born players displaying lower scores than players born in Q1 (*H* = 9.085, *p* = 0.028). In other sub-dimensions of physical self-concept, the proportions of Q1-born players tend to be higher than that of Q3- and Q4-born players, but these differences were not significant. When we verified for RAEs specific to age, no significant differences were observed. However, we observed some significant differences that support RAEs at the competitive level. For global physical self-concept, Q1 and Q2 players showed higher scores than those of Q3 and Q4 (*H* = 9.863, *p* = 0.020). Similar results were observed for perceived strength (*H* = 8.162, *p* = 0.043), and perceived sport competence tends to be higher among Q1 and Q2 players (*H* = 7.011, *p* = 0.072). In summary, there is a RAE in minor hockey, especially among competitive players. Birth quartile does not appear to cause disparities in attitudes toward physical activities but has an impact on general self-concept, perceived sport competence, and perceived strength.

### Discussion for Study 1

Results from study 1 confirm that the RAE is prevalent in Quebec's minor hockey. This is similar to Baker et al.'s (2010) study that showed that RAEs are prevalent in minor ice hockey. Interestingly, despite the prevalence of RAEs, they do not seem to be specific to an age group or a playing level. This result is also in line with Grondin et al. (1984), who showed that RAEs applied to all minor hockey age groups, from Atom (9 and 10 years old) to Midget (15 and 16 years old). However, in our data, there was no difference regarding birth distribution, which means that RAEs prevail from U13 to U18. However, our data do not allow us to confirm or discard the idea that birth distributions differ in each cohort. Using longitudinal data (each category of birth distribution) would be most relevant for testing whether RAEs fluctuate among specific cohorts and for testing whether sports attrition, which tends to begin at adolescence, is related to the RAE (Delorme et al., 2011). In addition to a possible attrition among older players, our younger participants were only 12 and 13 years old. At this age, boys have not quite reached the peak of puberty, which usually occurs 1 to 2 years later (Marshall and Tanner, 1970; Williams and Currie, 2000). The physical benefits associated with puberty (increase in strength, size, and power, among others) that may accentuate the bias that favors early-born players may not be maximized at this stage of their development, which may have reduced RAEs among different age groups (Musch and Grondin, 2001). In this regard, anthropometric measures and an assessment of the pubertal stage reached would be useful in future studies.

Results regarding psychosocial outcomes showed partial support for RAEs in minor ice hockey. First, we observed no significant results regarding attitudes toward hockey, sports, and exercise behaviors. From this perspective, our results showed that RAEs do not affect young hockey players' attitudes toward their sport and other kinds of physical activities, even when considering age group and playing level. However, we have to be cautious in our interpretation because it cannot be excluded that this absence of impact of the RAE on players' attitudes is caused by the strong and favorable attitudes toward ice hockey (e.g., a possible ceiling effect). In fact, players who had a negative attitude toward ice hockey may have already given up on the sport, as sport dropouts sometimes occur even before the beginning of adolescence.

Results regarding physical self-concept measures showed some interesting differences. Such results are somewhat congruent with past RAE research, albeit with quite small effect sizes and with slightly younger participants (Thompson et al., 2004; Kawata et al., 2017). In terms of measures of physical self-concept, our results showed a tendency in which Q3 players tend to show lower scores, but not Q4. In fact, in each measure of physical self-concept, Q4 players showed similar scores to those of Q1 players. Moreover, our results showed that RAEs were also significant according to the level of competition in which players evolve. Results from our study suggest that RAEs seemed to be present among competitive players on measures of physical self-concept (global), perceived sport competence, and perceived strength. This would mean that early-born players showed stronger perceptions of their physical competencies. Such small differences can be explained by several hypotheses. First, there is a possibility that players build their self-perceptions mostly by comparing themselves almost exclusively with teammates and opponents (Marsh, 1987), and, assuming that the player selection process is rigorous at the beginning of the season, there should be no major differences in participants' perceived competence over a season. A study focusing on young ice hockey players' experience showed that RAEs are indeed present, but that there are no significant differences in terms of minutes played or physical interactions with opponents between those born earlier and later in the year (Baker et al., 2010). In this regard, Baker concludes that it makes sense that players from the same team tend to develop similar perceptions of competence since they are similar in their physical stature and in their level of play, and have faced the same level of competition over a season. Even if we do not have anthropometrical data, our results suggest that, despite the presence of a RAEs, adolescent hockey players have very similar psychosocial attributes regardless of their birth quartile and level of play, with the noteworthy exception of perceived sport competence in the most competitive levels. Having equivalent self-concept scores between Q1 and Q4 players is congruent with the psychological hypothesis that suggests that late-born players develop strong psychological attributes to survive sports selection (Ashworth and Heyndels, 2007). Furthermore, it is possible that using a global sport competence physical self-concept subscale instead of a hockey-specific physical self-concept subscale has reduced the precision of competence assessment and, therefore, has underestimated the RAE on physical self-concept. While this point is beyond the scope of this study, it is relevant to point out that developing a hockey-specific perceived competence scale might help refine and identify key differences that could be related to players' hockey experience. Such a specific scale was developed by Forsman et al. (2016); they successfully created and validated a soccer-specific competence scale.

## Study 2: Prevalence and Performance in the CHL

### Sample

In study 2, we assessed the prevalence of the RAE in junior hockey and compared players born in the four different quartiles on multiple performance indicators.

Data were extracted from raw data files pick224.com^1^ This website provides databases extracted from official CHL game box scores and includes performance metrics that are not available on the CHL website. We selected players who participated in 2018–2019 in the CHL, which combines the 3 major junior leagues: Western Hockey League (WHL), Ontario Hockey League (OHL), and Quebec Junior Major Hockey League (QJMHL). The CHL is identified as the predominant league, providing over 32% of NHL players (Canadian Hockey League, 2019). Total sample consists of 1318 players distributed almost equally across leagues (WHL: *n* = 478; OHL: *n* = 433; QJMHL: *n* = 407). Mean age (± SD) was also similar across leagues (OHL = 18.31 ± 1.24 years; WHL = 18.3 ± 1.21 years; QJMHL = 18.43 ± 1.16 years).

### Measures

Birth quartile was calculated from raw data that were available in each database. For both studies, each birth date was coded into birth quartiles (Q1 to Q4), the same quartiles as the ones used in study 1. Body weight and height were used to verify if early-born players differ from late-born players. Based on past research in this field (Grondin and Trudeau, 1991), we expected no major differences on players' anthropometric profiles.

***Performance outcomes*** were assessed using metrics that were available in the data files. In the junior sample, we assessed players' performance with 4 performance metrics: (1) games played (*n*), (2) total points, (3) 5-on-5 points (goals + assists), and (4) power-play points. Such indicators were used in past research (Wattie et al., 2007) and are commonly used to reflect a player's utilization and his offensive contribution to his team.

### Statistical Analyses

We tested for the presence of RAEs by conducting cross-tabulation analyses and calculating chi-square (χ^2^) scores for the entire CHL sample. Same analyses were conducted with each league separately. In a way similar to that of study 1, we tested the distribution of births in quartiles using expected values from the 2001 distribution of births in Canada (median birth year; Statistique Canada, 2018). Secondly, we compared players' age and anthropometric data (height and weight) across birth quartiles by conducting one-way ANOVAs, with the Bonferroni correction for *post-hoc* analyses. To evaluate RAEs on performance indicators, we used quantile regressions to verify if RAEs tend to diminish on multiple performance indicators that were available in the CHL database. Compared to OLS regression, quantile regression is more flexible to non-normal data distributions (Hao and Naiman, 2007). Quantile regression is also used to compare subsamples and prevent selection bias that may be caused by an arbitrary smaller sample (Lê Cook and Manning, 2013). Regression analyses were conducted on the 25th, 50th, 75th, and 90th percentiles of each distribution. This technique was performed in the past to verify differences among NHL players' number of games played and total points (Fumarco et al., 2017). RAEs would be maintained if early-born players still performed better than the late-born players (at the 75th and 90th percentile). However, results from the Fumarco et al. (2017) study make it more difficult to confirm if a reversal of the RAE really prevails, especially because players' age varies more substantially at this level. From this perspective, it becomes most relevant to verify whether the same kind of pattern occurs at a younger age. In this regard, a stabilization of the RAE would occur if no significant differences in performance were observed (no significant regression coefficient in the 75th and 90th percentile), and a reversal of the RAE would occur when the performance of late-born players was better than that of early-born players.

### Results

#### Prevalence of RAEs in the CHL

Table 4 shows birth distribution across the three CHL leagues. Results suggest that the RAE is prevalent in the CHL, and more specifically in all three of the CHL leagues (χ^2^ > 85.00, *p* < 0.001), with significant deviations from Canada birth distribution for the year 2001. Effect sizes for each league were moderate to large (*W* > 0.45), suggesting a high level of disproportion between birth quartiles. We must specify that players from other nationalities (e.g., Europe, USA) are eligible to play in the CHL. A total of 232 CHL players (OHL = 41%; QJMHL = 28%; WHL = 31%) came from outside Canada, which represents 17% of the total sample. When considering players' birth country, RAEs remained significant, with a medium effect size (χ^2^ = 19.96, *p* < 0.001, *W* = 0.30)^2^

**Table 4:** Distribution of births in each quartile in the Canadian hockey league (CHL).

**Birth quartile**	**Total *N* (%) [Standardized residuals]**	**WHL *N* (%)**	**OHL *N* (%)**	**QJMHL *N* (%)**	**Birth quartiles in Canada 2001**
Q1	553 (41.6%) [218.5]	203 (42.5%) [83.5]	177 (40.9%) [68.8]	168 (41.3%) [66.3]	24%
Q2	383 (28.7%) [48.5]	130 (27.2%) [10.5]	126 (29.1%) [17.8]	122 (30.0%) [20.3]	26%
Q3	237 (18.5%) [−85.5]	89 (18.6%) [−30.5]	87 (20.1%) [−21.2]	68 (16.7%) [−33.7]	26%
Q4	145 (11.2%) [−181.5]	56 (11.7%) [−63.5]	43 (9.9%) [−65.2]	49 (12.0%) [−52.7]	24%
Total	1 318	478	433	407	N/A
χ^2^	274.194^*^	100.795^*^	90.076^*^	85.708^*^	N/A
Cohen's *W*	0.469	0.459	0.456	0.459	N/A

* *p < 0.001*.

#### Morphological Differences in the CHL

Results from anthropometric data, reported in Table 5, showed no significant differences regarding height and weight (*F*_height_ = 0.472, *p* = 0.702; *F*_weight_ = 0.368, *p* = 0.776), even if Q4 players tend to be taller. Such results mean that players who evolve in the CHL have similar morphological attributes at this stage of development.

**Table 5:** Anthropometric measures of CHL players as a function of birth quartile.

	**Birth quartile**				
**Measures**	**Total**	**Q1**	**Q2**	**Q3**	**Q4**
Height (cm)	181.3 ± 10.54	181.1 ± 10.81	181.5 ± 10.11	180.8 ± 10.71	182.0 ± 10.4
Weight (kg)	83.75 ± 7.23	83.79 ± 7.29	83.76 ± 7.17	84.14 ± 7.37	83.75 ± 7.23

#### RAEs and Performance Indicators

RAEs prevail for powerplay contribution (PP-pts) and the number of games played. Scores from regression analyses showed performance levels at the upper quartiles, which is associated with the best level of performance across each birth quartile. As shown in Table 6, analyses were conducted for the 75th and 90th percentiles. The most important RAEs were observed at percentile 90, which represents CHL's most performing players. For instance, Q1 and Q2 displayed more points than Q4 players, and Q2 outperformed Q4 players in powerplay points.

**Table 6:** Birth quartile and performance in the CHL: Results from quantile regression.

**Outcome**	**Birth quartile (with 95% Confidence Intervals)**				
	**Constant**	**Q1**	**Q2**	**Q3**	**Q4**
GP75	64 (62.94–65.06)	66^*^ (0.81–3.19)	66^*^ (0.75–3.25)	66^*^ (0.66–3.34)	64 (n/a)
GP90	68 (67.46–68.54)	68 (−0.61–0.61)	68 (−0.64–0.64)	68 (−0.69–0.69)	68 (n/a)
Pts75	38 (30.25–45.75)	40 (−6.37–10.74)	40 (−7.14–11.15)	42 (−5.83–13.83)	38 (n/a)
Pts90	52 (41.69–62.31)	62^**^ (−1.61–21.61)	64^**^ (−0.16–24.16)	63 (−2.06–24.06)	52 (n/a)
5 vs. 5 Pts75	28 (11.58–14.41)	28 (−0.69–2.68)	27 (−0.73–2.73)	31^***^ (−0.83–2.83)	28 (n/a)
5 vs. 5 Pts90	36 (20.14–23.86)	42 (−0.21–4.21)	41 (−1.29–3.29)	45^*^ (−0.42–4.42)	36 (n/a)
PPpts 75	6 (3.53–8.47)	8 (−0.78–4.78)	8 (−0.91–4.91)	11^***^ (−0.13–6.13)	6 (n/a)
PPpts 90	12 (8.75–15.25)	15 (−0.67–6.67)	16^***^ (0.16–7.84)	15 (−1.13–7.13)	12 (n/a)

* *Games Played (GP): Q1_75_-Q2_75_ > Q4_75_, p < 0.01*.

** *5 vs. 5 pts: Q1_90_ > Q4_90_; Q1_90_ Q2_90_ > Q4_90_, p < 0.10*.

*** *PP Pts: Q3_75_ > Q4_75_, p = 0.10; Q1_90_ > Q4_90_, p < 0.05; Q2_90_ > Q4_90_, p < 0.05; Q3_90_ > Q4_90_, p = 0.10*.

### Discussion for Study 2

Study 2 shows that RAEs in major junior Canadian hockey prevails across the country, as has been the case for more than 60 years (Wattie et al., 2007). RAEs have not declined at the major junior level (CHL), leading us to believe that early-born players are still prioritized in player selection in ice hockey. Such results are in line with those of Barnsley et al. (1985), obtained more than 35 years ago with OHL and WHL players. More recently, Nolan and Howell (2010) “revisited” RAEs in the CHL and showed that the Canadian hockey culture has not changed over the decades. Our results suggest that the ways to assess talent or potential have remained the same over time. From this perspective, such results are in line with Hancock's model (Hancock et al., 2013a), in which coaches and scouts are social agents who influence talent detection and team selection; consequently, they help maintain RAEs in elite hockey. Interestingly, our results showed that similar RAE patterns seem to prevail among non-Canadian players, who came into the CHL wishing to get more exposure and to increase their chances to be drafted in the NHL.

From a players' physical stature standpoint, we think the attributed value of having a strong physical stature remains important, which is in line with what was demonstrated by many authors. In fact, players' morphology has increased over the last 50 years (taller, heavier, stronger), which suggests an advantage for early-born players at younger levels (Montgomery, 2006; Quinney et al., 2008). The CHL showed no differences regarding stature of players regarding birth quartile, which is in line with past research on this topic (Grondin and Trudeau, 1991). Therefore, this suggests that late-born players who “survived” the selection process at the major junior level are possibly those who are more mature physically, despite their birth month (early developers). In addition, as the body size of NHL players seems to have plateaued over the last decade (Wendorf, 2015), the prototype of the small, fast, and skilled players has emerged in the mid-2010s NHL (Larkin, 2019). Professional hockey can be thought of as a “copycat league,” in which contenders tend to imitate the Stanley Cup champions' “recipe” for success. In this regard, we might, at first glance, find this recent shift of paradigm encouraging for late-born players who do not have the same “physical” advantage as their “older” teammates. However, this rejoicing might well be short-lived, as we are seeing a return to the norm of “big and fast” prototype players among recent Stanley Cup champions (Larkin, 2019), which gives an advantage to early-borns.

The second part of study 2 focused on performance outcomes. This issue recently raised some interest by proposing the hypothesis of a reversal of the RAE at later stages of athletic development (Gibbs et al., 2012). Such a hypothesis is plausible given that players who survive the unfavorable years may develop more resilience, character, and abilities, which serve them to perform better in the future. One such hypothesis was supported in other sports (McCarthy et al., 2016) and among established NHL players (Fumarco et al., 2017) by showing that late-born players performed at least as well as early-born players who were prioritized previously at the entry draft. Results from study 2 are partially consistent with the hypothesis of a reversal of RAEs at the CHL level. Our results showed that despite the fact that early-born players receive more exposure (e.g., games played, points), they do not perform substantially better than late-born players on a more global estimate of performance (e.g., total points and 5 vs. 5 points). This suggests a possible shift toward a reversal of RAEs at the major junior level.

Despite such observations, our analyses of the performance outcomes have their limitations. In study 2, we considered indicators related to playing time, offensive production, and puck possession. Many other indicators could have been considered and deserve more attention for future research. For instance, future studies should include additional indicators of players' performance (e.g., face offs, turnovers, shooting %, etc.). Such approaches are now more feasible with the emergence of artificial intelligence-based applications. In a more practical way, it would benefit major junior organizations' stakeholders (scouting staff, coaches, etc.) to be aware of the results of study 2. Late-born players have the potential to catch up at a later stage of their development. Consequently, stakeholders should consider ways to continue talent evaluation on a longer-term basis or could simply begin the evaluation process at a later stage (e.g., after 16 years old). This approach would be better adapted to long-term athlete development and sports expertise models (Balyi and Way, 2005).

## General Discussion

Results from the first part of this study shows that although the RAE in Canadian ice hockey has been identified almost 40 years ago, there is still a disproportionally high number of early-born players in Canadian hockey. The popularity of the sport combined with specific ice hockey tasks (physicality, skills, and gender) can also explain why RAEs are still strong over the years. However, we think that the policy regarding age categories (24 months per category) is the main reason that explains why RAEs are still prevalent in Canadian hockey. As the Lavoie et al. (2015) study showed, simply changing the cutoff dates had an immediate impact that would favor the “new” early-born players. In this regard, it is relevant to think about new age categories to diminish RAEs and offer equal opportunities for all players, beginning with avoiding 24-month-long categories (Grondin et al., 1984). Comparing the Canadian situation with the organization of minor and junior hockey leagues and talent identification in other countries would be an interesting avenue for future research and would provide further knowledge about the impacts of RAEs in ice hockey.

Study 1 was a first attempt to shed light on the potential psychological impacts of RAE at the minor hockey level. Beyond the simple analysis of prevalence, the present study set out to verify to what extent RAEs can affect different ice hockey players' experiences. According to study 1, RAEs do not seem to affect the enjoyment of playing ice hockey, but it has an impact on the development of physical self-perception, especially among the most competitive players. As Hancock's (2013) social agents model stipulates, decision makers, coaches, parents, and athletes themselves need to be informed and concerted about RAEs, in order to better understand the developmental aspects of hockey players, from the beginning to the end of an athletic “pathway.”

The second part of our study demonstrates that RAEs also prevail at the elite junior level in Canada, and possibly in other countries. As Wattie et al. (2015) suggest, a complex interaction involving the multiple facets of the game, combined with environmental factors, make the RAE a real concern, even at such high levels of competition. Interestingly, our analyses also demonstrated that there are RAEs among non-Canadian CHL players. Such results are congruent with those obtained with Czech (Bozděch and Zháněl, 2019) and Russian players (Bezuglov et al., 2020). Additional research should be conducted among CHL stakeholders (coaches, scouts, and managers), who are in charge of talent detection and recruitment. Such research would refine our understanding of the RAE occurrence at the elite level and could provide additional insights that would help to diminish discrimination against late-born players, some of whom are forgotten on the pathway toward elite hockey. The analyses of performance indicators showed that despite being less represented, late-born players have performances comparable to those of early-born players, suggesting a potential reversal of the RAE. These results highlight the need to further examine the outcomes on the players themselves, but also by taking more into account the perceptions of coaches, scouts, and agents on the criteria they use when observing the most promising talents in ice hockey.

## Conclusion and Future Directions

This study provides an updated overview of the RAE in the field of Canadian ice hockey. Despite being a subject that has generated scientific interest over the last 40 years, the RAE is still a predominant phenomenon in Canada's national sport. In summary, despite the presence of RAEs in the sport, late-born players who have gotten through the selection process seem to have similar psychosocial (in minor hockey) and morphological (in major junior) profiles. Educating coaches and raising awareness about RAEs in sports is still necessary to prevent sports dropout and provide equal opportunities for players to manifest their talent at different levels. Decision makers should also think about offering late-born players more exposure opportunities, namely, by playing against (or with) early-born players or by introducing them to different kinds of events (showcases, testing sessions, etc.). Despite its contribution to better understanding RAEs, this study has its limitations and suggests interesting research avenues for the future. Longitudinal designs that include players' anthropometric measures would give a better estimate of how the RAE phenomenon takes place in the early stages of sports development (e.g., 12 years old). A more in-depth scrutinization of coaches' and scouts' perspectives about RAEs and talent would also help to understand how it could be approached in a long-term perspective. Finally, cooperation and dialogue with sport federations would certainly help to identify key actions that would allow young athletes to continue to develop not only as athletes but also as healthy individuals.

## Data Availability Statement

The original contributions presented in the study are included in the article/Supplementary Material, further inquiries can be directed to the corresponding author/s.

## Ethics Statement

The studies involving human participants were reviewed and approved by CER-17-240-08-01.10. Written informed consent to participate in this study was provided by the participants' legal guardian/next of kin.

## Author Contributions

JL supervised redaction of the manuscript. He was involved in relation (Study 1 and Study 2), statistical analyses and literature review. VH was mostly involved in the literature review process, writing the manuscript (Study 1 and general discussion) and took part to statistical analyses (Study 1). FT was involved in redaction (discussions, S1, S2 and general), and provide expertise in the problem statement. SG participated in manuscript redaction (problem statement, discussion, analyses) and provide additional background (literature review) in the theoretical background of both studies. All authors confirm having participated to the preparation of this manuscript.

## Conflict of Interest

The authors declare that the research was conducted in the absence of any commercial or financial relationships that could be construed as a potential conflict of interest.

## Footnotes

^1^Data extracted from the website https://pick224.com.

^2^Significant RAEs were observed in QJMHL, but not in the OHL and WHL. Further analyses regarding rules governing the draft of European and American players are needed.
